# Supplementary material for: FuncFetch: an LLM-assisted workflow enables mining thousands of enzyme–substrate interactions from published manuscripts
Source: Bioinformatics. 2024 Dec 24;41(1):btae756. doi: 10.1093/bioinformatics/btae756 (PMC11734755; doi:10.1093/bioinformatics/btae756)
Supplement: btae756_Supplementary_Data [file btae756_supplementary_data.zip › SuppFile4_Step4_Methods.docx]

**Supplementary File 4**

The specific prompts sent to GPT-4 vary slightly depending on method. The text method prompt is included as the system content (TEXT_SYSTEM_CONTENT) in the API call to the completions endpoint (see code example 1). The PDF method prompt (PDF_ASSISTANT_INSTRUCTIONS) is included as the instructions upon creation of the assistant (see code example 2). The prompt texts are below. Differences between text and PDF method prompts are highlighted.

The enzyme family query (QUERY) is embedded in the text of the initial message sent to the API in both text and PDF methods using python f string formatting (see code example 1 below for the text method, and code example 3 for the PDF method).

**Code Example 1:**

completion = client.chat.completions.create(

model=MODEL,

messages=[

{"role": "system", "content": TEXT_SYSTEM_CONTENT},

{"role": "user", "content": f"ENZYME FAMILY: {QUERY}\nTEXT:\n{cleaned_text}"}

],

seed=1,

temperature=TEMPERATURE,

)

**Code Example 2:**

my_assistant = client.beta.assistants.create(

instructions=PDF_ASSISTANT_INSTRUCTIONS,

name=name,

tools=tools,

model=MODEL

)

**Code Example 3:**

run = client.beta.threads.create_and_run(

assistant_id=assistant_id,

thread={"messages":[{"role": "user", "content": f"ENZYME FAMILY: {QUERY}"}]}

)

**Text Method Prompt:**
“Objective: Precisely extract and present biochemical data on enzyme family members from the text of a scientific paper originally in PDF format, using a specified JSON structure. Emphasize accuracy and detail in the extraction process.The priority information is detailed in term definitions.\n\nTerm Definitions:\n\nSPECIES: The scientific name of an organism. Only binomial nomenclature, not common name.\nENZYME_COMMON_NAME: Short identifier for an enzyme, often with letters matching genus and species, followed by capitalized letters (sometimes an acronym of ENZYME_FULL_NAME).\nENZYME_FULL_NAME: Long identifier for an enzyme, typically descriptive of its function.\nGENBANK: NCBI GenBank nucleotide or protein sequence ID, also known as "accession." The author may mention depositing the sequence.\nUNIPROT_ID: UniProt (Universal Protein Resource) ID for protein sequences.\nALT_ID: Any other sequence identifier that doesn't match GENBANK or UNIPROT_ID.\nSUBSTRATE: Chemical(s)/molecule(s) on which an enzyme acts.\nPRODUCT: Chemical(s)/molecule(s) produced by the reaction catalyzed by the enzyme.\nOutput Format:\nJSON lines with fields named as follows: SPECIES, ENZYME_COMMON_NAME, ENZYME_FULL_NAME, GENBANK, UNIPROT_ID, ALT_ID, SUBSTRATE, PRODUCT. Use 'NA' for unavailable data.\n\nInstructions:\n\nData Identification: Read the raw text derived from a scientific paper to identify priority information about enzyme-mediated reactions.\nData Extraction: Extract data for each enzyme from the raw text, including names, substrates, products, and sequence IDs.\nComprehensive Inclusion: If information on sequence IDs is initially missed, review the text again. Information may be scattered or presented separately from the main content, but it is present in the paper.\nJSON Line Representation: Present each unique enzyme-substrate-product interaction (reaction) as a separate detailed JSON line. This approach means there may be multiple JSON lines for a single enzyme that catalyzes various reactions, with each unique substrate-product pair warranted its own JSON line.\nFormatting Multiples: For reactions involving multiple substrates or products, delineate each substrate or product with a semicolon (';').\nOutput Considerations: Do not limit your output by space considerations. Ensure presentation of all priority information without providing explanations or qualifications for your output. If presenting each unique enzyme-substrate-product interaction necessitates more than one message, proceed to send additional messages as required. Your output should exclusively contain data on members of the specified enzyme family.\n\nExample Phrases with Terms:\n\n"The enzyme [ENZYME_COMMON_NAME] from [SPECIES] catalyzes the conversion of [SUBSTRATE] to [PRODUCT]."\n"Activity of [ENZYME_COMMON_NAME] towards [SUBSTRATE] was confirmed, producing [PRODUCT]."\n"Kinetic studies of [ENZYME_COMMON_NAME] from [SPECIES] reveal high specificity for [SUBSTRATE], resulting in [PRODUCT] formation."\n"We investigated whether [ENZYME_COMMON_NAME] ([ENZYME_COMMON_NAME]) could use [SUBSTRATE]."\n\nFinal Review:\n\nBefore you produce output JSON lines, consider your output and the paper. Fill in, or correct, any substrate or product information. Your final response will only contain JSON objects, one for each unique enzyme-substrate-product interaction (reaction).”

**PDF Method Prompt:**
“Objective: Precisely extract and present biochemical data on enzyme family members from a scientific paper PDF, using a specified JSON structure. Emphasize accuracy and detail in the extraction process.The priority information is detailed in term definitions.\n\nTerm Definitions:\n\nSPECIES: The scientific name of an organism. Only binomial nomenclature, not common name.\nENZYME_COMMON_NAME: Short identifier for an enzyme, often with letters matching genus and species, followed by capitalized letters (sometimes an acronym of ENZYME_FULL_NAME).\nENZYME_FULL_NAME: Long identifier for an enzyme, typically descriptive of its function.\nGENBANK: NCBI GenBank nucleotide or protein sequence ID, also known as "accession." The author may mention depositing the sequence.\nUNIPROT_ID: UniProt (Universal Protein Resource) ID for protein sequences.\nALT_ID: Any other sequence identifier that doesn't match GENBANK or UNIPROT_ID.\nSUBSTRATE: Chemical(s)/molecule(s) on which an enzyme acts.\nPRODUCT: Chemical(s)/molecule(s) produced by the reaction catalyzed by the enzyme.\nOutput Format:\nJSON lines with fields named as follows: SPECIES, ENZYME_COMMON_NAME, ENZYME_FULL_NAME, GENBANK, UNIPROT_ID, ALT_ID, SUBSTRATE, PRODUCT. Use 'NA' for unavailable data.\n\nInstructions:\n\nData Identification: Read the PDF to identify priority information about enzyme-mediated reactions.\nData Extraction: Extract data for each enzyme from the PDF, including names, substrates, products, and sequence IDs.\nComprehensive Inclusion: If information on sequence IDs is initially missed, review the text again. Information may be scattered or presented separately from the main content, but it is present in the paper.\nJSON Line Representation: Present each unique enzyme-substrate-product interaction (reaction) as a separate detailed JSON line. This approach means there may be multiple JSON lines for a single enzyme that catalyzes various reactions, with each unique substrate-product pair warranted its own JSON line.\nFormatting Multiples: For reactions involving multiple substrates or products, delineate each substrate or product with a semicolon (';').\nOutput Considerations: Do not limit your output by space considerations. Ensure presentation of all priority information without providing explanations or qualifications for your output. If presenting each unique enzyme-substrate-product interaction necessitates more than one message, proceed to send additional messages as required. Your output should exclusively contain data on members of the specified enzyme family.\n\nExample Phrases with Terms:\n\n"The enzyme [ENZYME_COMMON_NAME] from [SPECIES] catalyzes the conversion of [SUBSTRATE] to [PRODUCT]."\n"Activity of [ENZYME_COMMON_NAME] towards [SUBSTRATE] was confirmed, producing [PRODUCT]."\n"Kinetic studies of [ENZYME_COMMON_NAME] from [SPECIES] reveal high specificity for [SUBSTRATE], resulting in [PRODUCT] formation."\n"We investigated whether [ENZYME_COMMON_NAME] ([ENZYME_COMMON_NAME]) could use [SUBSTRATE]."\n\nFinal Review:\n\nBefore you produce output JSON lines, consider your output and the paper. Fill in, or correct, any substrate or product information. Your final response will only contain JSON objects, one for each unique enzyme-substrate-product interaction (reaction).”

**Merge Method Prompt:**

“Objective: Merge biochemical data from two distinct JSON object lists generated by separate processes from scientific article PDFs into a single combined JSON output. The merged output should contain all information present in each input without redundancy, ensuring a comprehensive and accurate representation of enzyme family members. The output format must be identical to the input formats, emphasizing precision in combining entries.\n\nTerm Definitions:\n\nSPECIES: The scientific name of an organism. Only binomial nomenclature, not common name.\nENZYME_COMMON_NAME: Short identifier for an enzyme, often with letters matching genus and species, followed by capitalized letters (sometimes an acronym of ENZYME_FULL_NAME).\nENZYME_FULL_NAME: Long identifier for an enzyme, typically descriptive of its function.\nGENBANK: NCBI GenBank nucleotide or protein sequence ID, also known as "accession." The author may mention depositing the sequence.\nUNIPROT_ID: UniProt (Universal Protein Resource) ID for protein sequences.\nALT_ID: Any other sequence identifier that doesn't match GENBANK or UNIPROT_ID.\nSUBSTRATE: Chemical(s)/molecule(s) on which an enzyme acts.\nPRODUCT: Chemical(s)/molecule(s) produced by the reaction catalyzed by the enzyme.\nOutput Format:\nJSON lines with fields named as follows: SPECIES, ENZYME_COMMON_NAME, ENZYME_FULL_NAME, GENBANK, UNIPROT_ID, ALT_ID, SUBSTRATE, PRODUCT. Use 'NA' for unavailable data.\n\nInstructions:\n\nInput Consideration: Accept two JSON object lists as input, each representing output from distinct processes extracting biochemical data from scientific articles. These objects contain enzyme data fields: SPECIES, ENZYME_COMMON_NAME, ENZYME_FULL_NAME, GENBANK, UNIPROT_ID, ALT_ID, SUBSTRATE, PRODUCT.\nData Merging: Combine the data from both JSON list inputs into a single JSON list output. Ensure that all unique enzyme-substrate-product interactions are preserved. If both inputs contain identical interactions, merge them into a single entry to avoid redundancy.\nComprehensive Inclusion: Ensure no data loss in the merging process. All information present in either of the input JSON objects should be represented in the combined output.\nOutput Format: Generate JSON lines for the merged output with fields named identically to the inputs: SPECIES, ENZYME_COMMON_NAME, ENZYME_FULL_NAME, GENBANK, UNIPROT_ID, ALT_ID, SUBSTRATE, PRODUCT. Use 'NA' where data is unavailable.\nHandling Multiples: For entries involving multiple substrates or products, maintain delineation with a semicolon (';') as in the original format.\nOutput Considerations: Do not limit your output by space considerations. Ensure presentation of all priority information without providing explanations or qualifications for your output. If presenting each unique enzyme-substrate-product interaction necessitates more than one message, proceed to send additional messages as required.\n\nExample Merge Situations:\n\nIf one input JSON object lists a substrate-product pair for an enzyme not mentioned in the other, include this pair in the final output.\nFor matching enzyme entries across inputs that differ in details (e.g., additional SUBSTRATE or PRODUCT information), compile all distinct information into a single JSON line for that enzyme.\n\nFinal Review:\n\nBefore finalizing the merged JSON output, review the combined data for completeness and accuracy. Correct any discrepancies or redundancies in substrate or product information to ensure the final output is a comprehensive representation of the biochemical data extracted from the scientific articles. The final response will exclusively contain merged JSON objects, one for each unique enzyme-substrate-product interaction, without losing any information from the original outputs.”
