# Supplementary material for: FuncFetch: an LLM-assisted workflow enables mining thousands of enzyme–substrate interactions from published manuscripts
Source: Bioinformatics. 2024 Dec 24;41(1):btae756. doi: 10.1093/bioinformatics/btae756 (PMC11734755; doi:10.1093/bioinformatics/btae756)

**Supplementary File 8:** Analysis of hallucinations. **(A)** First example of messages presenting information from model weights. The pdf method describes searching the document for mentions of the target enzyme families, but only returns entries with the target names and no additional information. In this case, the text method returns a set of correct enzyme activities, but with no relation to information in the text. The information presented is similar to the output when the same model is asked about the enzyme family in question with no added context (see **8C**). **(B)** Second example of messages presenting information from model weights. In this case, both pdf and text methods present similar information to the output when the same model is asked about the enzyme family in question with no added context (see **8C**). **(C)** Screenshot of ChatGPT response with GPT-4o model when prompted to describe UDP-glycosyltransferases and their substrates.

**A. Message 1:**

pdf_method | 10.1042/bj2940231

```json

{

"SPECIES": "NA",

"ENZYME_COMMON_NAME": "NA",

"ENZYME_FULL_NAME": "UDP-glycosyltransferase",

"GENBANK": "NA",

"UNIPROT_ID": "NA",

"ALT_ID": "NA",

"SUBSTRATE": "NA",

"PRODUCT": "NA"

}

{

"SPECIES": "NA",

"ENZYME_COMMON_NAME": "UGT",

"ENZYME_FULL_NAME": "UDP-glycosyltransferase",

"GENBANK": "NA",

"UNIPROT_ID": "NA",

"ALT_ID": "NA",

"SUBSTRATE": "NA",

"PRODUCT": "NA"

}

{

"SPECIES": "NA",

"ENZYME_COMMON_NAME": "NA",

"ENZYME_FULL_NAME": "UDP-glucuronosyltransferase",

"GENBANK": "NA",

"UNIPROT_ID": "NA",

"ALT_ID": "NA",

"SUBSTRATE": "NA",

"PRODUCT": "NA"

}

```

The provided json lines capturate elements from all the search results regarding all variations of UDP-glucosyltransferase, UGT and UDP-glucuronosyltransferase. See the sources for additional nuanced information.

--------------------

text_method | 10.1042/bj2940231

```json

{

"SPECIES": "Homo sapiens",

"ENZYME_COMMON_NAME": "UGT1A1",

"ENZYME_FULL_NAME": "UDP-glucuronosyltransferase 1A1",

"GENBANK": "NA",

"UNIPROT_ID": "P22309",

"ALT_ID": "NA",

"SUBSTRATE": "bilirubin",

"PRODUCT": "bilirubin diglucuronide"

}

{

"SPECIES": "Homo sapiens",

"ENZYME_COMMON_NAME": "UGT1A6",

"ENZYME_FULL_NAME": "UDP-glucuronosyltransferase 1A6",

"GENBANK": "NA",

"UNIPROT_ID": "P19224",

"ALT_ID": "NA",

"SUBSTRATE": "acetaminophen",

"PRODUCT": "acetaminophen glucuronide"

}

{

"SPECIES": "Mus musculus",

"ENZYME_COMMON_NAME": "UGT2B1",

"ENZYME_FULL_NAME": "UDP-glucuronosyltransferase 2B1",

"GENBANK": "NA",

"UNIPROT_ID": "Q8R1N6",

"ALT_ID": "NA",

"SUBSTRATE": "testosterone",

"PRODUCT": "testosterone glucuronide"

}

{

"SPECIES": "Rattus norvegicus",

"ENZYME_COMMON_NAME": "UGT2B7",

"ENZYME_FULL_NAME": "UDP-glucuronosyltransferase 2B7",

"GENBANK": "NA",

"UNIPROT_ID": "P06129",

"ALT_ID": "NA",

"SUBSTRATE": "morphine",

"PRODUCT": "morphine-3-glucuronide; morphine-6-glucuronide"

}

{

"SPECIES": "Homo sapiens",

"ENZYME_COMMON_NAME": "UGT2B15",

"ENZYME_FULL_NAME": "UDP-glucuronosyltransferase 2B15",

"GENBANK": "NA",

"UNIPROT_ID": "P54855",

"ALT_ID": "NA",

"SUBSTRATE": "androsterone",

"PRODUCT": "androsterone glucuronide"

}

{

"SPECIES": "Homo sapiens",

"ENZYME_COMMON_NAME": "UGT1A3",

"ENZYME_FULL_NAME": "UDP-glucuronosyltransferase 1A3",

"GENBANK": "NA",

"UNIPROT_ID": "P35503",

"ALT_ID": "NA",

"SUBSTRATE": "estrone",

"PRODUCT": "estrone glucuronide"

}

{

"SPECIES": "Homo sapiens",

"ENZYME_COMMON_NAME": "UGT1A4",

"ENZYME_FULL_NAME": "UDP-glucuronosyltransferase 1A4",

"GENBANK": "NA",

"UNIPROT_ID": "P22310",

"ALT_ID": "NA",

"SUBSTRATE": "trifluoperazine",

"PRODUCT": "trifluoperazine glucuronide"

}

{

"SPECIES": "Homo sapiens",

"ENZYME_COMMON_NAME": "UGT1A9",

"ENZYME_FULL_NAME": "UDP-glucuronosyltransferase 1A9",

"GENBANK": "NA",

"UNIPROT_ID": "O60656",

"ALT_ID": "NA",

"SUBSTRATE": "propofol",

"PRODUCT": "propofol glucuronide"

}

{

"SPECIES": "Homo sapiens",

"ENZYME_COMMON_NAME": "UGT2B4",

"ENZYME_FULL_NAME": "UDP-glucuronosyltransferase 2B4",

"GENBANK": "NA",

"UNIPROT_ID": "P06128",

"ALT_ID": "NA",

"SUBSTRATE": "chenodeoxycholic acid",

"PRODUCT": "chenodeoxycholic acid glucuronide"

}

{

"SPECIES": "Homo sapiens",

"ENZYME_COMMON_NAME": "UGT2B17",

"ENZYME_FULL_NAME": "UDP-glucuronosyltransferase 2B17",

"GENBANK": "NA",

"UNIPROT_ID": "Q9Y4X8",

"ALT_ID": "NA",

"SUBSTRATE": "testosterone",

"PRODUCT": "testosterone glucuronide"

}

```

~~~~~~~~~~~~~~~~~~~~

**B. Message 2:**

~~~~~~~~~~~~~~~~~~~~

pdf_method | 10.1007/978-1-62703-758-7_11

```json

{

"SPECIES": "NA",

"ENZYME_COMMON_NAME": "UGT1A1",

"ENZYME_FULL_NAME": "UDP-glucuronosyltransferase 1A1",

"GENBANK": "NA",

"UNIPROT_ID": "NA",

"ALT_ID": "NA",

"SUBSTRATE": "Bilirubin; Estradiol",

"PRODUCT": "Bilirubin glucuronide; Estradiol glucuronide"

}

{

"SPECIES": "NA",

"ENZYME_COMMON_NAME": "UGT1A4",

"ENZYME_FULL_NAME": "UDP-glucuronosyltransferase 1A4",

"GENBANK": "NA",

"UNIPROT_ID": "NA",

"ALT_ID": "NA",

"SUBSTRATE": "Tamoxifen; Dihydrotestosterone",

"PRODUCT": "Tamoxifen glucuronide; Dihydrotestosterone glucuronide"

}

{

"SPECIES": "NA",

"ENZYME_COMMON_NAME": "UGT2B7",

"ENZYME_FULL_NAME": "UDP-glucuronosyltransferase 2B7",

"GENBANK": "NA",

"UNIPROT_ID": "NA",

"ALT_ID": "NA",

"SUBSTRATE": "Zidovudine",

"PRODUCT": "Zidovudine glucuronide"

}

{

"SPECIES": "NA",

"ENZYME_COMMON_NAME": "UGT1A9",

"ENZYME_FULL_NAME": "UDP-glucuronosyltransferase 1A9",

"GENBANK": "NA",

"UNIPROT_ID": "NA",

"ALT_ID": "NA",

"SUBSTRATE": "Propofol",

"PRODUCT": "Propofol glucuronide"

}

{

"SPECIES": "NA",

"ENZYME_COMMON_NAME": "UGT1A6",

"ENZYME_FULL_NAME": "UDP-glucuronosyltransferase 1A6",

"GENBANK": "NA",

"UNIPROT_ID": "NA",

"ALT_ID": "NA",

"SUBSTRATE": "Serotonin",

"PRODUCT": "Serotonin glucuronide"

}

{

"SPECIES": "NA",

"ENZYME_COMMON_NAME": "UGT2B15",

"ENZYME_FULL_NAME": "UDP-glucuronosyltransferase 2B15",

"GENBANK": "NA",

"UNIPROT_ID": "NA",

"ALT_ID": "NA",

"SUBSTRATE": "S-oxazepam",

"PRODUCT": "S-oxazepam glucuronide"

}

```

--------------------

text_method | 10.1007/978-1-62703-758-7_11

```json

{

"SPECIES": "Homo sapiens",

"ENZYME_COMMON_NAME": "UGT1A1",

"ENZYME_FULL_NAME": "UDP-glucuronosyltransferase 1A1",

"GENBANK": "NA",

"UNIPROT_ID": "P22309",

"ALT_ID": "NA",

"SUBSTRATE": "bilirubin; estradiol",

"PRODUCT": "bilirubin glucuronide; estradiol-3-glucuronide"

}

{

"SPECIES": "Homo sapiens",

"ENZYME_COMMON_NAME": "UGT1A3",

"ENZYME_FULL_NAME": "UDP-glucuronosyltransferase 1A3",

"GENBANK": "NA",

"UNIPROT_ID": "P35503",

"ALT_ID": "NA",

"SUBSTRATE": "estradiol",

"PRODUCT": "estradiol-3-glucuronide"

}

{

"SPECIES": "Homo sapiens",

"ENZYME_COMMON_NAME": "UGT1A4",

"ENZYME_FULL_NAME": "UDP-glucuronosyltransferase 1A4",

"GENBANK": "NA",

"UNIPROT_ID": "P22310",

"ALT_ID": "NA",

"SUBSTRATE": "tamoxifen; dihydrotestosterone",

"PRODUCT": "tamoxifen glucuronide; dihydrotestosterone glucuronide"

}

{

"SPECIES": "Homo sapiens",

"ENZYME_COMMON_NAME": "UGT1A6",

"ENZYME_FULL_NAME": "UDP-glucuronosyltransferase 1A6",

"GENBANK": "NA",

"UNIPROT_ID": "P19224",

"ALT_ID": "NA",

"SUBSTRATE": "serotonin",

"PRODUCT": "serotonin glucuronide"

}

{

"SPECIES": "Homo sapiens",

"ENZYME_COMMON_NAME": "UGT1A9",

"ENZYME_FULL_NAME": "UDP-glucuronosyltransferase 1A9",

"GENBANK": "NA",

"UNIPROT_ID": "O60656",

"ALT_ID": "NA",

"SUBSTRATE": "entacapone; propofol; 4-MU",

"PRODUCT": "entacapone glucuronide; propofol glucuronide; 4-MU glucuronide"

}

{

"SPECIES": "Homo sapiens",

"ENZYME_COMMON_NAME": "UGT2B7",

"ENZYME_FULL_NAME": "UDP-glucuronosyltransferase 2B7",

"GENBANK": "NA",

"UNIPROT_ID": "P16662",

"ALT_ID": "NA",

"SUBSTRATE": "zidovudine; morphine; androsterone",

"PRODUCT": "zidovudine glucuronide; morphine-6-glucuronide; androsterone glucuronide"

}

{

"SPECIES": "Homo sapiens",

"ENZYME_COMMON_NAME": "UGT2B15",

"ENZYME_FULL_NAME": "UDP-glucuronosyltransferase 2B15",

"GENBANK": "NA",

"UNIPROT_ID": "P54855",

"ALT_ID": "NA",

"SUBSTRATE": "S-oxazepam",

"PRODUCT": "S-oxazepam glucuronide"

}

{

"SPECIES": "Homo sapiens",

"ENZYME_COMMON_NAME": "UGT2B17",

"ENZYME_FULL_NAME": "UDP-glucuronosyltransferase 2B17",

"GENBANK": "NA",

"UNIPROT_ID": "Q9Y4X8",

"ALT_ID": "NA",

"SUBSTRATE": "NA",

"PRODUCT": "NA"

}

```

~~~~~~~~~~~~~~~~~~~~

**C.**


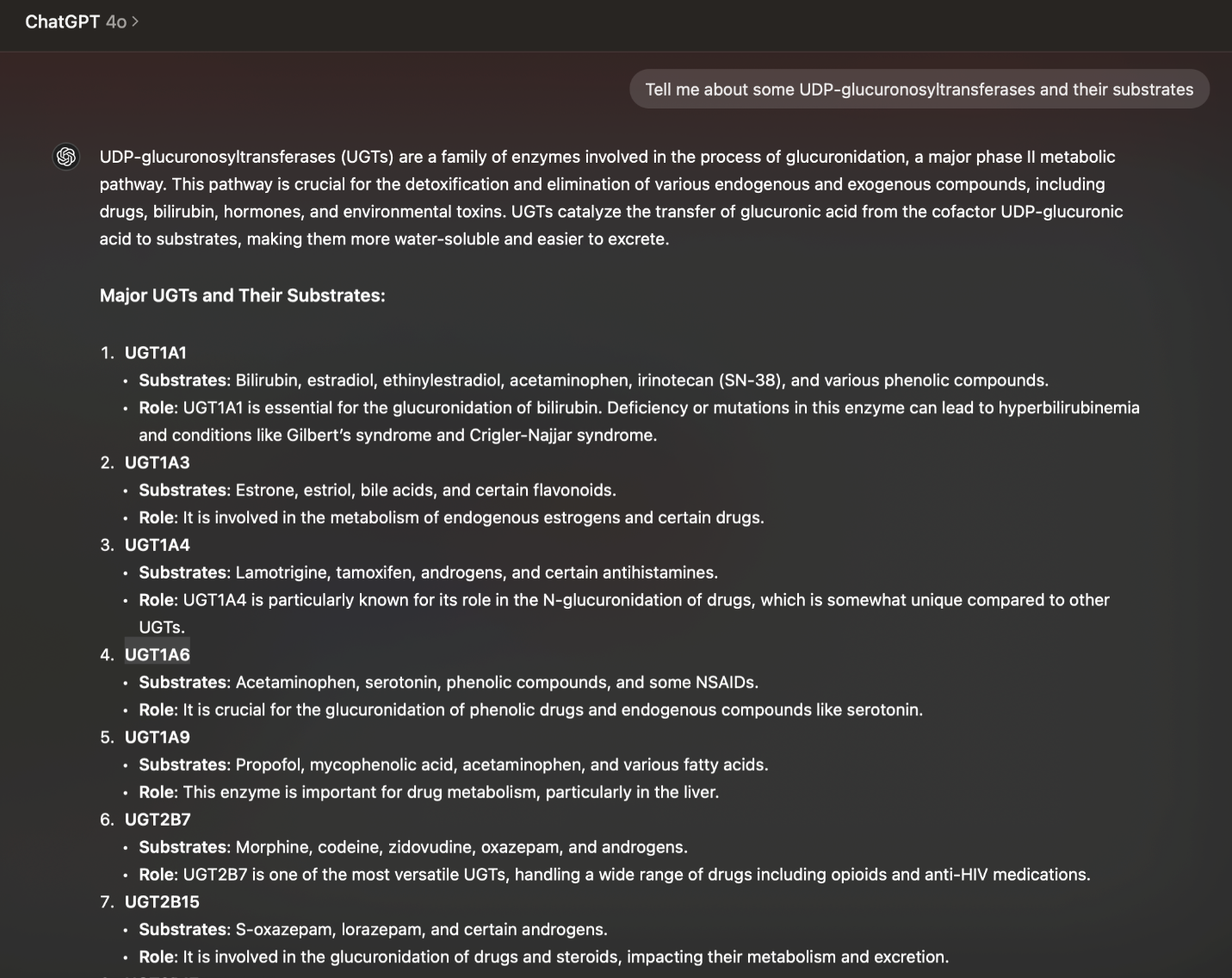

Supplement: btae756_Supplementary_Data [file btae756_supplementary_data.zip › SuppFile8_Hallucinations.docx]
